# Groundnut Bud Necrosis Virus Encoded NSm Associates with Membranes via Its C-Terminal Domain

**DOI:** 10.1371/journal.pone.0099370

**Published:** 2014-06-11

**Authors:** Pratibha Singh, Shantinath S. Indi, Handanahal S. Savithri

**Affiliations:** 1 Department of Biochemistry, Indian Institute of Science, Bangalore, India; 2 Department of Microbiology and Cell Biology, Indian Institute of Science, Bangalore, India; University of California, Riverside, United States of America

## Abstract

Groundnut Bud Necrosis Virus (GBNV) is a tripartite ambisense RNA plant virus that belongs to serogroup IV of Tospovirus genus. Non-Structural protein-m (NSm), which functions as movement protein in tospoviruses, is encoded by the M RNA. In this communication, we demonstrate that despite the absence of any putative transmembrane domain, GBNV NSm associates with membranes when expressed in *E. coli* as well as in *N. benthamiana*. Incubation of refolded NSm with liposomes ranging in size from 200–250 nm resulted in changes in the secondary and tertiary structure of NSm. A similar behaviour was observed in the presence of anionic and zwitterionic detergents. Furthermore, the morphology of the liposomes was found to be modified in the presence of NSm. Deletion of coiled coil domain resulted in the inability of *in planta* expressed NSm to interact with membranes. Further, when the C-terminal coiled coil domain alone was expressed, it was found to be associated with membrane. These results demonstrate that NSm associates with membranes via the C-terminal coiled coil domain and such an association may be important for movement of viral RNA from cell to cell.

## Introduction

The primary infection caused by the entry of viruses in plants, following mechanical injury to the cell wall and plasma membrane, is mostly confined to a single cell. The progeny viruses need to move to other sites in the plants from the initial infection site to establish generalized infection. The cell wall is impervious to viral penetration, therefore, the channel connecting adjacent cells, namely the plasmodesmata, facilitates the cell to cell movement of viruses. The size exclusion limit of plasmodesmata is such that it does not allow the movement of the virus or viral genome from one cell to other. Plant viruses encode specialised proteins called movement proteins (MP) which assist the transport of the virus or viral genome [1] from cell to cell. In addition to MP, viral movement is dependent on other viral ancillary proteins and host factors residing in the plasmodesmata [2]. It was shown that Tobacco mosaic virus (TMV) MP is associated with endomembrane system and has two hydrophobic helices that span endoplasmic reticulum [3]. On the other hand the MPs of other viruses such as Alfaalfa mosaic virus (AMV) form tubules that connect the adjacent cells through which the virus can move [4]. The AMV MP and other MPs from viruses belonging to different genera are shown to be associated with membrane *via* their transmembrane domain or hydrophobic regions [3], [5]–[11].

Groundnut bud necrosis virus (GBNV) also called Peanut bud necrosis virus (PBNV) is a member of tospovirus genus which belongs to *bunyaviridae* family [12]. The family *bunyaviridae* is comprised of five genera that include *Orthobunyavirus, Hantavirus, Nairovirus, Phlebovirus and Tospovirus* [13]. Tospovirus is the only genus that infects plants and has again been divided into four serogroups based on serological relationships and nucleocapsid protein sequence homology with Tomato spotted wilt virus (TSWV), the type member of this genus [12]. The virions are enveloped, quasi-spherical particles of 80–120 nm in diameter that encapsidate three negative stranded RNA genomes. The L, M and S RNA encode for RNA dependent RNA polymerase (RdRP), glycoproteins G1 and G2 and nucleocapsid protein (N) in the virion complementary sense respectively. Apart from this M and S RNA also encode for two non-structural proteins NSm and NSs respectively in the sense orientation [12].

Among tospoviruses reported from India, GBNV is the predominant virus causing the maximum loss and has been well characterized [14]. GBNV belongs to serogroup IV and shares 29% identity with N Protein of TSWV [15]. The N and NSm genes of several isolates of GBNV, from different locations in India, infecting Leguminosae, Solanaceae and Cucurbitaceae plants, have been sequenced and shown to belong to serogroup IV [16]–[18]. The NSs protein from GBNV Tomato isolate from Karnataka (GBNV-To (K)) is a multifunctional enzyme and has NTPase as well as 5′phosphatase activity [19]. NSs from TSWV and GBNV are also shown to be suppressors of post transcriptional gene silencing [20]–[22]. The N Protein of GBNV binds to RNA and can be phosphorylated by *Nicotiana benthamiana* plant crude sap [23].

There are only two plant virus genera, tospoviruses and Rhabdoviruses that have the unique membrane envelopes surrounding the nucleocapsids and encapsidate their RdRps [24]. The mechanism of assembly and movement of these viruses is complex and is, therefore, interesting. NSm is the protein which facilitates the movement of tospoviruses. It forms tubular structures in insect cells and protoplasts [25], [26]. Further, TSWV NSm was shown to interact with host proteins such as DNAj like protein, other plasmodesmata residing proteins and viral N Protein [27]. The domains involved in tubule formation and viral movement have been identified by alanine scanning mutagenesis to be from G^19^–S^159^ and G^209^–V^283^ [28] respectively. These domains are large and need further delineation.

In the present study, NSm gene of GBNV-To (K) was cloned and overexpressed in *Escherichia coli*. The recombinant NSm was found to be associated with *E. coli* membranes. The refolded GBNV NSm devoid of these membranes was shown to bind to liposomes resulting in structural alteration of the interacting partners. Further, transiently expressed GBNV NSm in *Nicotiana benthamiana* was also found to be associated with membranes that could not be solubilized under a variety of conditions. The deletion of C-terminal coiled coil domain resulted in the inability of NSm to localize to cell membrane.

## Materials and Methods

All the *E. coli* strains used in this study, the affinity purification kits and other molecular biological reagents were purchased from Novagen–EMD4 Biosciences (USA). The oligonucleotide primers used for amplification of the wild-type NSm gene were custom made by Sigma Chemicals.

Primers used in this study:

- NSm Sense Primer: 5′ CTAGCTAGCCATATGTCTCGCTTTTCTAACG 3′
- NSm Antisense Primer: 5′ CGGAATTCCGTTATATTTCAAGAAGATTATCC 3′
- NSm CΔ65 antisense primer: 5′ GCGGAATTCTTAAATGGGAACTACATC 3′
- NSmC79 sense primer: 5′ GGGGCTAGCCCTATAGCTGCTG 3′

### *In silico* Analysis of Secondary Structure of NSm

Prediction of the presence of a transmembrane domain was carried out using HMMTOP server, http://gcat.davidson.edu/DGPB/kd/kyte-doolittle.htm and http://www.cbs.dtu.dk/services/TMHMM, http://www.sbc.su.se/~miklos/DAS server.

To find out the unfolded and folded domains, fold index server (http://bip.weizmann.ac.il/fldbin/findex) was used. The coiled coil domain was identified by coils prediction server (http://toolkit.tuebingen.mpg.de/pcoils).

### Cloning, Overexpression and Purification

Total RNA was isolated from infected leaf material by triazol method [29]. RT-PCR was carried out using NSm specific primers designed on the basis of GBNV M RNA sequence (accession no. AY871097), to amplify the NSm gene. The PCR product was cloned in pRSETC at NheI and EcoRI site such that the overexpressed recombinant NSm (rNSm) would have N-terminal hexa histidine tag. pRSETC-Nsm clone was transformed into BL21pLysS *E. coli* cells. A single colony was inoculated to LB medium and the expression of NSm was induced by the addition of IPTG to 0.3 mM. The expression was analysed by SDS-PAGE [30]. The recombinant NSm was purified by Ni-NTA chromatography under denaturing conditions and refolded as described in the manufacturer’s protocol. The identity of the purified protein was confirmed by western blot analysis using polyclonal antibodies to purified NSm at 1∶10,000 dilution.

### Purification of NSm by Sucrose Density Gradient Centrifugation

The NSm clone was transformed into *E. coli* BL21 (DE3) pLys S cells and the protein was expressed upon induction with 0.3 mM IPTG for four hours. The cell pellet was resuspended in the lysis buffer (50 mM Tris/HCl pH 8.0, 200 mM NaCl, 0.1% Triton X-100) and sonicated. After sonication the cell lysate was spun at 10,700 g and the supernatant was subjected to ultracentrifugation at 100000 g for 3 hours. The pellet thus formed was again resuspended in lysis buffer and was again subjected to low speed centrifugation. The supernatant was then layered on to 10%–40% sucrose density gradient, ultracentrifuged at 100000 g for 3 hours. Fractions (2 ml) were collected and analysed by SDS PAGE.

### Liposome Preparation

Multilamellar liposomes were prepared by the hydration method [31]. 10 mg of egg phosphotidylcholine (PC) was dissolved in CHCl_3_. The mixture was dried as a thin film under a stream of nitrogen. The lipid film was then stored under vacuum for more than 6 hours to remove the traces of organic solvent. The dry lipid film was hydrated by the addition of 50 mM Tris/HCl buffer (pH 8.0) containing 200 mM NaCl and then vigorously vortexed for 1 min to produce multilamellar vesicles (MLVs). The MLV suspensions were frozen and thawed for three cycles. A single freeze-thaw cycle consisted of freezing for 1 min at liquid nitrogen temperature (−196°C) and thawing for 1 min in a water bath at 40°C.

### Membrane/Liposome Flotation Assays

The floatation assay was performed as described earlier [32]. Egg PC MLVs were prepared as described above. Protein (1 mg/ml in Tris/HCl pH 8.0 and 200 mM NaCl) samples and liposomes (10 mg/ml in 50 mM Tris/HCl pH 8.0 and 200 mM NaCl) were mixed together and incubated at 37°C for 30 min. Sucrose density gradient was set up as follows: 1.6 ml of 85% sucrose and 300 µl liposome-rNSm complex prepared as above was mixed to make a final concentration of 71.9% sucrose. This mixture was placed at the bottom of 12 ml swinging bucket centrifuge tube and the mixture was overlaid with 7 ml of 60% sucrose followed by 3 ml of 10% of sucrose. It was then centrifuged at 200000 g for 18 hours at 4°C. After centrifugation, the tube was pierced at the bottom and fractions (2 ml) were collected. Membrane/membrane-protein complex floats at the interface of 10% and 60% sucrose solution (fraction 5 and 6). Soluble protein remains at the bottom of the gradient (fraction 1). Each fraction was loaded on the SDS PAGE and western blot analysis was performed using anti NSm antibodies (1∶10,000).

### Analytical Ultracentrifugation

Purified refolded NSm (1 mg/ml) was subjected to analytical ultracentrifugation in presence and absence of 300 µl of liposomes in Beckman XLA model ultracentrifuge at 100000 g. The absorbance at 280 nm was measured as a function of distance migrated by the protein/protein-liposome complex.

### Circular Dichroism Spectroscopy

rNSm purified by sucrose density gradient and refolded NSm in 25 mM Tris/HCl pH 8.0 containing 100 mM NaCl were used to record the far UV CD spectra in a Jasco-814 polarimeter. The ellipticity was monitored at 25°C from 200 to 250 nm. A scan speed of 50 nm/min, path length of 0.2 cm, band width of 1 nm and response time of 1 sec were used. The spectra were averaged for 3 scans and corrected with buffer blanks.

### Fluorescence Spectroscopy

Fluorescence experiments were performed using a Perkin-Elmer LS5S luminescence spectrometer (Perkin-Elmer, Waltham, MA, USA). The intrinsic fluorescence emission of rNSm (100 µg/ml) was monitored from 300 nm to 450 nm upon excitation at 280 nm. To monitor tryptophan fluorescence only, NSm was excited at 295 nm. The wavelength at which maximum emission is observed provides an indication of the extent to which the aromatic amino acid residues are exposed to the solvent. The spectra were averaged for three scans and corrected with buffer blanks.

### Transmission Electron Microscopy

The transmission electron microscopy of samples was carried out using JEOL-100 CX II transmission electron microscope (TEM) at 80 kV at 27000X. The average diameter of the liposomes was measured in seven different frames in presence and absence of NSm and the changes in the size were measured.

### Binary Constructs

NSm was cloned into binary vectors pEAQHT [33] at SmaI site to obtain pEAQNSm. The deletion mutant of NSm namely CΔ65 were generated by cloning the respective PCR products into pEAQ at SmaI site. The clone was designated as pEAQNSmCΔ65. Further the C-terminal domain was cloned into pEAQ vector and the clone was designated as pEAQC79.

### Plant Material, Growth Conditions and Agroinfiltration Procedures

*N. benthamiana* plants grown in a growth chamber under an18 h light/6 h dark photoperiod at 25°C were used for all agroinfiltration experiments. Leaves were infiltrated with *Agrobacterium tumefaciens* strain C58C1 essentially as described earlier [34]. Strain C58C1 was maintained on LB agar containing rifampicin (50 µg/ml). This medium was modified by adding kanamycin to a final concentration of 50 µg/ml in order to maintain the transformed C58C1 agrobacteria harbouring the binary pEAQ constructs. Plasmids were transformed into C58C1 by electroporation method [35]. For agroinfiltration, suspensions of transformed C58C1 bacteria were adjusted to an OD_600 _nm of 0.6 in MES buffer (10 mM MgCl_2_, 10 mM MES, pH 5.6), and acetosyringone was added to a final concentration of 150 µM. Bacterial suspensions were then maintained at 25°C for 2 to 3 hours. Infiltrations were conducted by gently pressing a 1 ml disposable syringe without needle to the abaxial surface of fully expanded leaves that were approximately 2.5 cm wide at the midleaf and slowly depressing the plunger. A sufficient amount of bacterial suspension was used to completely infiltrate the leaves and give a water-soaked appearance. Following agroinfiltration, plants were maintained in the growth chamber for at least 72 hours.

### Immunoblot Analysis of Proteins Expressed in Agroinfiltrated Leaves

Agroinfiltrated *N. benthamiana* leaves (2 g) were homogenised in 5 ml of buffer (25 mM HEPES pH 7.4). The crude extracts (30 µl) were mixed with 2X SDS loading dye, boiled for 5 minutes at 95°C, separated on 12% SDS PAGE [30], electroblotted on to nitrocellulose membranes and subjected to western blot analysis using antiNSm antibodies. The crude extracts was also analysed using membrane floatation assay discussed earlier [32].

*N. benthamiana* leaves (5 g) were homogenized in 25 mM HEPES pH 7.4 buffer containing 5 mM MgCl_2_ and centrifuged at 100 g for 5 minutes. The supernatant was centrifuged at 10700 g for 20 min to generate S10 (supernatant) and P10 (pellet) fraction. The pellet was resuspended in 100 mM Na_2_CO_3_ (pH 11) or different concentration of salts or Triton X100. All the resulting fractions were subjected to SDS PAGE and subsequently analysed by western blotting for analysis of the solubility properties of membrane bound NSm.

## Results

### Purification of NSm Overexpressed in *E. coli* Using Sucrose Density Gradient Centrifugation and its Biophysical Characterization

**Figure 1:**
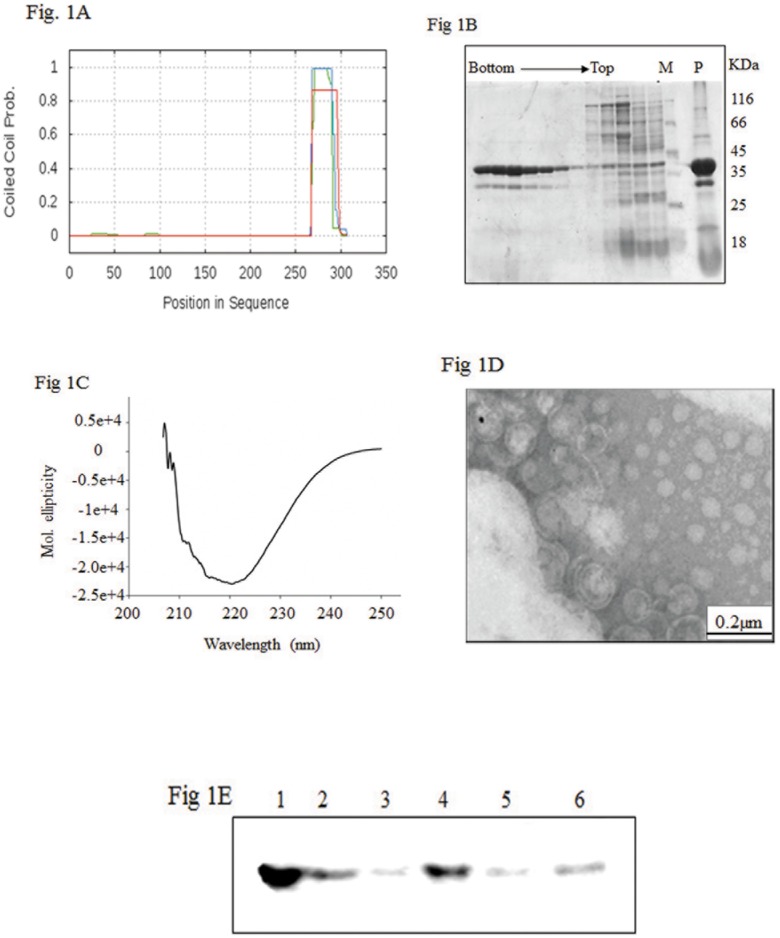
Purification of rNSm by sucrose density gradient centrifugation and its characterization. (**A**) *In silico* analysis of NSm: Plot of coiled coil Probability showing the putative C-terminal coiled coil domain in NSm. (**B**) Purification of rNSm: SDS PAGE of fractions (2 ml) collected after sucrose density gradient (10–40%) ultracentrifugation of the soluble fraction of NSm overexpressed in *E. coli.* Lane M indicates the molecular mass markers used, lane P represents the pellet (insoluble) fraction of NSm overexpressed in *E. coli.* (**C**) CD spectrum of NSm: The CD spectrum of NSm (200 µg) purified by sucrose density gradient centrifugation was recorded using Jasco-815 spectropolarimeter. The buffer used was 25 mM Tris/HCl pH 8, 100 mM NaCl. (**D**) TEM image of NSm: The NSm purified by sucrose density gradient after negative staining with 1% Uranyl acetate at 27,000X in a JEOL-100 CX TEM. (**E**) Membrane floatation assay of NSm: The crude lysate of NSm overexpressed in *E. coli* was subjected to membrane floatation. Lane 1 is the bottom fraction containing 71.9% sucrose, lanes 2 and 3 correspond to 60% sucrose, lane 4 and 5 are the fractions at interface of 60% and 10% sucrose (membrane fractions) and Lane 6 is top fraction containing 10% sucrose.

The putative region of NSm that might interact with membranes was predicted by *in silico* analysis of the NSm sequence using several different servers. No transmembrane domains were predicted to be present in the sequence of NSm. However, a coiled coil domain was predicted for the C- terminal 50 amino acid residues when COILS server (http://embnet.vital-it.ch/software/COILS_form.html) was used (Figure 1A).

In order to establish the membrane association characteristics of NSm, the GBNV-(To) K NSm gene was cloned in pRSET C vector between NheI and EcoRI sites and overexpressed in *E. coli* strain BL21 (DE3) pLysS. The overexpressed protein was analysed for solubility. A large fraction of the protein was found in the insoluble fraction (Figure 1B, lane P) and only a small fraction was seen in the soluble fraction. Attempts to purify NSm from the soluble fraction by Ni-NTA chromatography were unsuccessful as the fraction did not bind to Ni-NTA beads. Many recombinant viral MPs are known to aggregate and form inclusion bodies [3]. It is possible that the soluble NSm exists in an aggregated form and therefore it is not bound to the beads efficiently. In order to test this possibility, the soluble fraction was subjected to sucrose density gradient centrifugation. Fractions (2 ml) were collected and 30 µl from each fraction was analysed by SDS-PAGE. As shown in Figure 1B, the NSm (37 kDa) entered the gradient and was found between 30 and 40% of the sucrose. A minor contaminant of slightly lower molecular weight (∼30 KDa) band was also observed. However, the identity of this protein is not clear. The fractions containing NSm were pooled, dialyzed against 50 mM Tris/HCl buffer pH 8.0 containing 200 mM NaCl and used for further characterization.

The secondary structure of recombinant NSm was analysed by recording the far-UV CD spectrum. The recombinant NSm purified as described above showed a negative ellipticity peak at 222 nm (Figure 1C) that indicated the presence of alpha helix probably present as coiled coil structure [36]. The analysis of the spectrum using K2D2 software suggested that NSm is predominantly (81%) an alpha helical protein. Recently, it was shown that *Sesbania Mosaic Virus (SeMV)* MP is also a helical protein which interacts with its CP [37]. Other helical proteins well characterized for their role in movement are Tobacco mosaic virus (TMV) MP [38], TGBp1 of hordeivirus [39] and P7 of carmovirus [8].

### *E. coli* Expressed NSm is Associated with Membrane

As shown in Figure 1B, the soluble fraction of the *E. coli* expressed NSm sedimented between 30–40% sucrose, suggesting that it could have formed large aggregates [40]. In order to confirm the aggregated nature of the protein, transmission electron microscopy was performed. It was observed that the purified recombinant NSm appeared as large MLVs (Figure 1D). The possibility of presence of triton X100 micelles was ruled out by the fact that the sizes of such micelles are very small ranging from 28 Å to 100 Å [41]. But the sizes of MLVs were in range of 250 nm. These results suggested that MLVs were formed due to association of NSm with *E. coli* membranes. To confirm this, the crude *E. coli* lysate obtained after induction and expression of NSm was subjected to membrane floatation assay. The protein is expected to float or cofractionate along with membrane between 60% and 10% sucrose. Figure 1E shows the western blot analysis of fractions obtained after ultracentrifugation at 200000 g for 18 hours. As evident, the unbound aggregated NSm moved to the bottom (71.9% sucrose) (Figure 1E lane1). However substantial amount of NSm was found at the interface of 60% and 10% sucrose (Figure 1E, lane 4 and 5) and a small amount of NSm was seen in the top 10% sucrose (lane 6) suggesting that some of the NSm was memebrane associated.

### Refolded NSm Binds to Egg PC Derived Liposomes

**Figure 2:**
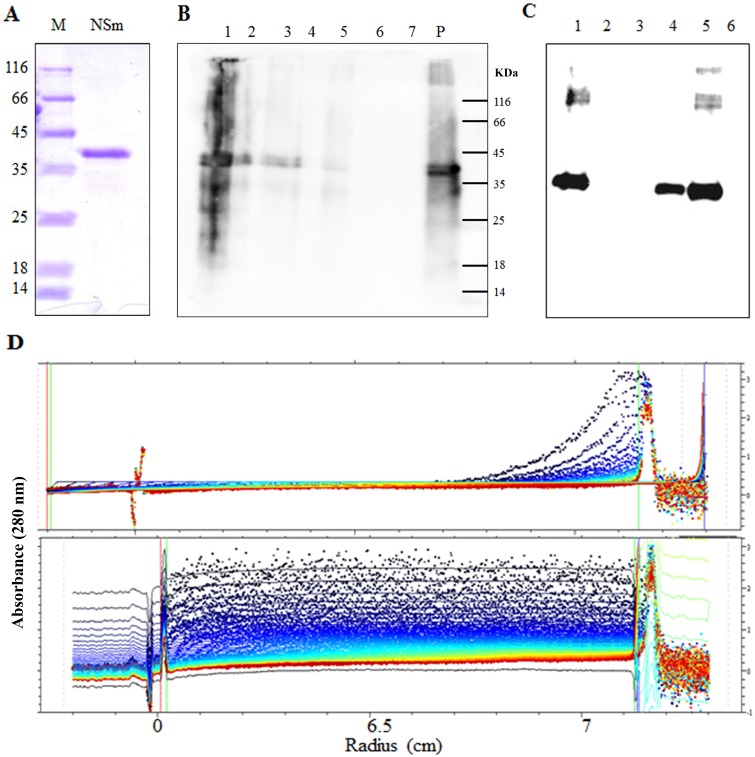
Purification of NSm under denaturing condition and properties of refolded NSm. (**A**) SDS PAGE of NSm purified by Ni-NTA affinity chromatography under denaturing condition and refolded by step wise dialysis: Lane M: Molecular mass markers, lane NSm: puirified NSm after step wise dialysis, (**B and C**) Refolded NSm binds to egg PC derived liposome: Membrane floatation assay of refolded NSm in the absence (B) and presence (C) of egg PC derived liposomes. Lane 1: bottom fraction containing 71.9% sucrose, Lane 2 and 3: fraction containing 60% sucrose, Lane 4 and 5: interface fractions of 60% and 10% sucrose (membrane fraction), lane 6: Top fraction (10% sucrose). (**D**) Sedimentation velocity profile of refolded NSm: 200 µg of Refolded NSm was subjected to analytical ultracentrifugation in presence (500 µl) and absence of liposomes. The absorbance at 280 nm is plotted as function of distance migrated by the protein/protein- liposome complex Top panel: Sedimentation profile of NSm alone, Bottom panel: Sedimentation profile of NSm-liposome complex.

Liposomes have been extensively used to study the membrane protein interaction and its impact on the biochemical function of the protein. In order to show the membrane association of NSm *in vitro*, it was purified under denaturing conditions and refolded. The refolded NSm was analysed by SDS PAGE and a single band (37 KDa) was found corresponding in size to the expected molecular mass of NSm (Figure 2A). Identity of the protein was confirmed by Western blot analysis using anti NSm antibody and peptide finger printing (data not shown).

To explore the association of refolded NSm with liposomes, membrane floatation assay was performed. Mixture of purified refolded NSm and liposome was placed at the bottom of the sucrose gradient and subjected to membrane floatation assay. Upon ultracentrifugation, the liposomes float up through the sucrose density gradient due to buoyancy [42]. As shown in Figure 2C, unbound aggregated protein remained at the bottom of gradient (lane 1) and some of the NSm appeared in lanes 4 and 5 the interphase of 60% and 10% sucrose As a control refolded NSm alone was subjected to membrane floatation assay and it was found to remain at the bottom (71.9% sucrose) and pellet fraction (Figure 2B). This suggests that refolded NSm can associate with liposomes.

Refolded NSm was also subjected to analytical ultracentrifugation in the presence and absence of liposomes. The rate of sedimentation of refolded NSm was drastically reduced in presence of liposomes (Figure 2D, bottom panel) as compared to its sedimentation in the absence of liposomes (Figure 2D, top panel). The density of liposomes is lower as compared to protein, so the sedimentation velocity of refolded NSm upon binding to liposomes decreased. The apparent sedimentation coefficient of NSm-liposome complex was 40S with RMSD of 0.006. However, refolded NSm was an aggregate, hence pelleted very fast. The sedimentation coefficient could not be calculated as the sedimentation profile was heterogeneous. The sedimentation rate was measured at 280 nm, at this wavelength liposomes alone do not show any absorbance. These experiments demonstrate that refolded NSm has the ability to associate with membranes.

### Refolded NSm Acquires Secondary and Tertiary Structure in Presence of Liposomes, SDS and other Detergents

**Figure 3:**
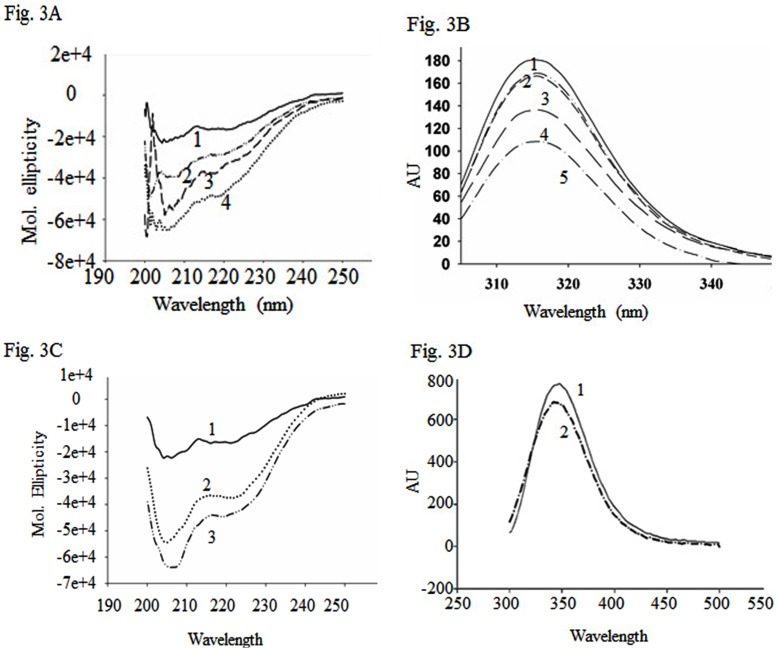
Effect of liposomes and SDS on secondary and tertiary structure of NSm. (**A**). The Far-UV CD spectra of refolded NSm (200 µg/ml) in the presence and absence of liposomes: (1) CD spectra of NSm alone, (2)–(4) CD spectrum of NSm in presence of 10, 50, 100 µl of liposomes respectively, (**B**) Fluorescence spectra of refolded NSm (100 µg/ml) in presence of liposomes: Fluorescence spectra were recorded upon excitation at 295 nm. (1) Fluorescence spectrum of NSm alone, (2), (3), (4) and (5) Fluorescence spectrum of NSm incubated with 5 µl, 10 µl, 20µl and 50 µl of liposomes respectively. (**C**) Far UV CD spectra in the presence SDS: curves 1, 2 and 3 represent the CD spectrum of (200 µg/ml) of NSm in presence of 0, 5 and 15 mM SDS respectively, (**D**) Fluorescence spectra of refolded NSm in presence of SDS: NSm was incubated with 5 mM SDS for 30 minutes and the fluorescence spectrum was recorded upon excitation at 280 nm. (1) Fluorescence spectrum of NSm (100 µg/ml) alone, (2) Fluorescence spectrum of NSm (100 µg/ml) in the presence of 5 mM SDS.

To further elucidate the structural changes in the refolded NSm caused by liposomes, far UV CD spectra were recorded in the presence and absence of liposomes. It was observed that there was an increase in negative ellipticity in the presence of liposome at 222 nm and 208 nm (Figure 3A) suggesting that refolded NSm gains alpha helical structure in the presence of liposomes. The interaction between liposomes and refolded NSm was also monitored by intrinsic fluorescence measurements. NSm has five tryptophan residues. Bioinformatics analysis suggested that three of these are in the random coil regions. The intrinsic fluorescence spectrum of refolded NSm (100 µg/ml) was recorded from 300 to 450 nm upon excitation at 295 nm. As shown in Figure 3B, refolded NSm exhibited emission maximum at 341 nm indicating that some of the tryptophan residues could be in a polar environment. Further, the fluorescence intensity decreased upon addition of lipid vesicles, although there was no change in the wavelength of emission maximum. These results suggest that binding of liposomes to NSm might result in quenching of fluorescence of exposed aromatic residues.

Detergents like SDS (sodium dodecyl sulphate) act as membrane analogues at low concentrations. Therefore, the far UV CD spectra of refolded NSm were recorded at low concentrations of SDS (5 and 15 mM). As shown in Figure 3C, refolded NSm gains significant secondary structure in presence of SDS. Further, there was a decrease in the fluorescence intensity at 343 nm upon addition of 5 mM SDS (Figure 3D) as observed earlier with liposomes (Figure 3B).

Different detergents were also used at a concentration of 5 mM to determine their effect on refolded NSm. Non-ionic (TWEEN20, NP40, Triton X100) detergents were not able to change the secondary structure of the protein significantly (data not shown) whereas anionic detergents like sodium deoxycholate, sodium sarcosine and zwittor ions like CHAPS resulted in the gain of secondary structure of refolded NSm as observed with SDS (data not shown).

### Refolded NSm Alters the Liposome Size and Surface

**Figure 4:**
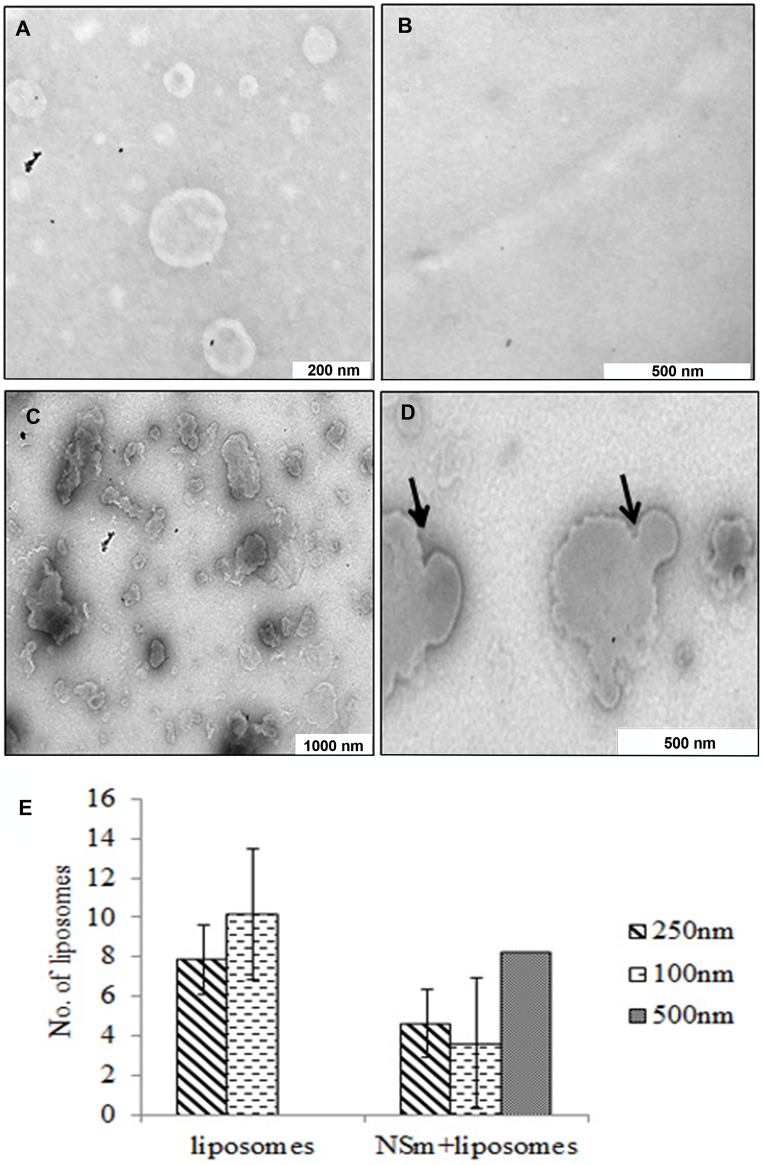
TEM images of liposomes showing membrane alteration in the presence of refolded NSm. (**A**) liposome alone. (**B**) NSm alone and (**C**) Liposomes incubated with refolded NSm. (**D**) Close up view of fused liposomes in presence of NSm (indicated by arrows). (**E**) The graph showing the the number of liposomes with average size of 250 nm and 500 nm in presence of NSm as compared to liposome alone. The error bars represent the error calculated from measurements on 7 independent frames.

In order to deduce the morphological changes in the liposome when incubated with NSm, the samples were visualised by transmission electron microscopy. As shown in Figure 4A, egg PC derived liposomes were largely spherical with 200–250 nm in diameter. When these liposomes were incubated with refolded NSm, the size of liposomes increased, they were flattened and irregular in shape (Figure 4C and D). Quantitation of the number of liposomes in the 250 nm range (Figure 4E) clearly demonstrated that number was reduced from thirty to six in the presence of NSm in seven independent frames. Further there was a concomitant increase in the number of liposomes in the 500 nm range as against none in the absence of NSm. This suggests that refolded NSm may alter the membrane curvature leading to clustering of the liposomes. NSm alone (Figure 4B) did not show the presence of vesicles confirming that refolded NSm is not associated with membranes.

### NSm Expressed *In planta* is also Membrane Associated through C-terminal Coiled Coil Region

**Figure 5:**
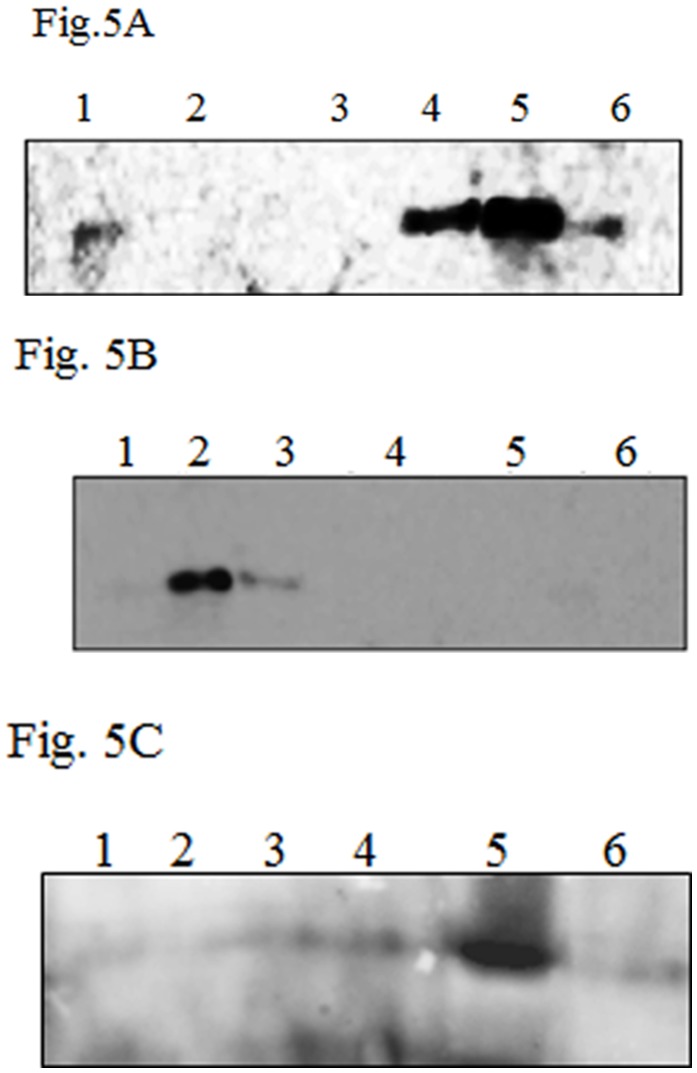
Immunoblot analysis of *N. benthamiana* leaves infiltrated with C58C1 transformed with pEAQ-NSm. (A) western blot analysis of crude extract of infiltrated leaves obtained after centrifugation at 12000 g. Supernatant (S) and pellet (P) (**B**), (**C**) and (**D**) Membrane floatation assay of *In planta* expressed NSm (B), NSmCΔ65 (C) and C-terminal coiled coil domain (C79 (D): lane 1: fraction with 71.9% sucrose, Lanes 2 and 3 are the fractions having 60% sucrose lanes 4 and 5 are the fraction at interphase of 60% and 10%, lane 6: fraction having 10% sucrose.

In order to confirm the membrane association of NSm, western blot analysis was performed after cell fractionation of *N. benthamiana* leaves infiltrated with Agrobacteria harbouring binary vectors with or without NSm gene. The western blot (Figure 5A) analysis using anti-NSm antibody was performed with the pellet and soluble fractions of the proteins extracted from leaves 72 hours post infiltration. A large fraction of NSm was present in the pellet fraction after centrifugation at 12000 g and a small fraction of NSm was present in supernatant which also pelleted upon centrifugation at 1,00,000 g (data not shown). To confirm further that *in* *planta* expressed NSm is also membrane associated membrane floatation assay was performed with the pellet fractions obtained after centrifugation at 12000 g as described earlier. As shown in Figure 5A, unlike *E. coli* expressed NSm, most of the i*n* *planta* expressed NSm was found in the membrane fractions (lanes 4 and 5) with a small amount in the 71.9% sucrose (lane 1) and 10% sucrose (lane 6).

In order to delineate the region responsible for membrane association, C-terminal 65 amino acids comprising coiled coil domain was deleted. pEAQ NSm CΔ65 was transformed into Agrobacterium and infiltrated separately into *N. benthamiana* leaves. The leaves were homogenised three days post infiltration and then subjected to membrane floatation assay. As shown in Figure 5B, NSm CΔ65 did not cofractionate with the membrane fraction. This suggests that NSm interacts with membrane via C-terminal coiled coil domain. In order to confirm that the C-terminal coiled coil domain is responsible for interaction with the membrane, the C-terminal coiled coil domain alone (pEAQ NSm C79) was expressed in *N. benthamiana* transiently. The infiltrated leaves were subjected to membrane floatation assay after 72 hours post infiltration, as apparent from Figure 5C, the coiled coil domain alone was able to associate with membrane further confirming that this domain could interact directly with the membrane.

### NSm is Tightly Associated with Membrane

**Figure 6:**
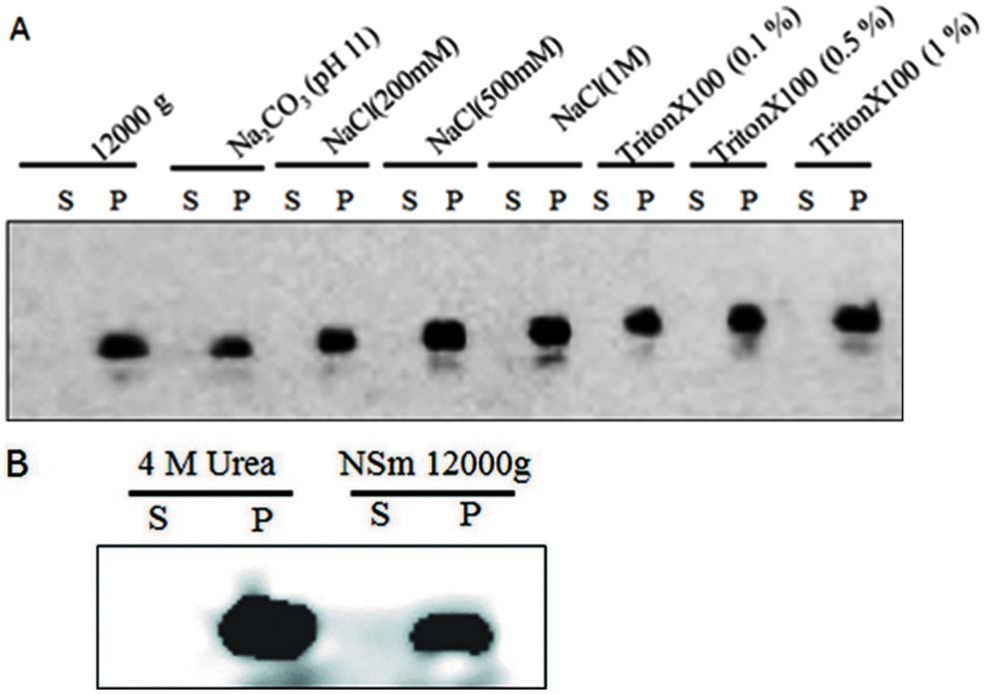
NSm is tightly associated with membranes. The crude extract of *N. benthamiana* leaves (1 ml each) infiltrated with transformed C58C1 containing pEAQNSm, obtained 60 h post infiltration was treated with 100 mM Na_2_CO_3_ (A), NaCl (0.2–1 M) (B) 4 M Urea (C) and Triton X100 (0.1–1%) (D) and centrifuged at 12,000 g. The supernatant (S) and pellet (P) fractions were subjected to 12% SDS PAGE followed by western blot analysis with anti NSm antibodies and detected by ECL.

To distinguish between the luminal and membrane associated NSm, 12000 g pellet fraction of NSm was treated with 100 mM Na_2_CO_3_ pH. 11 [9], [43] which is reported to open the microsomes [44] and thereby release the protein trapped in luminal fraction. Even after the treatment with Na_2_CO_3_, the protein was present in the pellet fraction (Figure 6A) suggesting that NSm is not present in the luminal fraction of microsomes. To determine whether NSm is integral or peripheral membrane protein, the 12000 g pellet fraction was treated with 1 M NaCl (Figure 6A). High salt causes the masking of charges and hence weakens the ionic strength required for peripheral membrane protein interaction. Such a treatment did not affect the solubility of NSm and it remained in the pellet fraction. Urea, a chaotropic agent, has the ability to release peripheral membrane protein into soluble fraction. But NSm remained in the pellet fraction (Figure 6B) associated with membrane even after treatment with 4 M urea. Similarly, Triton X100 (1%) could not solublize NSm (Figure 6A). These results suggest that NSm is membrane associated *in vivo* and that it could be an integral membrane protein [45] or it might form large aggregates that are not soluble in Na_2_CO_3,_ Triton X100, 1 M NaCl or 4 M urea.

## Discussion

There are several viral movement proteins of 30 K super family which have transmembrane domains. GBNV being a negative single stranded RNA virus is expected to have different strategy for viral movement. In this family, virions have RdRps as their structural constituent and are enveloped. Bioinformatic analysis of NSm did not reveal any putative transmembrane domain. However, a C-terminal coiled coil domain could be predicted (Figure 1A).

GBNV NSm shares 62% similarity with TSWV NSm and TSWV NSm was shown to form tubular structure in protoplasts as well as near plasmodesmata in infected plants. There are a number of movement proteins of different viruses known to form tubular structures in protoplasts and insect cell lines including TMV MP [46], [47]. The constituents of tubules and their functions are still unclear.

The results presented in this paper indicate that NSm interacts strongly with bacterial membrane as well as artificial membrane. The TEM images of NSm purified under non denaturing condition indicate the formation of MLVs (Figure 1D). Some of the MPs when expressed *in planta*, form vesicle like structures. For example, it has been demonstrated in Potato mop top virus TGB2 and TGB3 associates with endoplamic reticulum and colocalizes as motile granules by its incorporation into ER derived vesicles [48]. The potato virus X TGBp1 also associates with ER derived vesicles during virus infection [49].

The membrane floatation assay showed that the purified NSm was largely aggregated and some of the protein was associated with the membrane (Figure 1E). Several MPs are shown to aggregate as observed in the case of TMV MP [3]. *In silico* secondary structure predictions of NSm suggest the presence of coiled coil C-terminal domain (Figure 1A). The biological role of coiled coil domain is diverse. Such domains are shown to play an important role in formation of filaments in fibrous proteins and in oligomerization in tropomyosin [50]. Yet another function of coiled coil domain is membrane fusion as shown in the case of haemagglutin of Influenza virus [51] as well as in SNARE proteins [52], [53]. The TEM images of liposome also show that in presence of NSm, the size of liposomes are doubled (Figure 4C and D). The increase in size of the membrane vesicles could be the result of the membrane fusion mediated by the coiled coil domain.

Further, NSm was purified by Ni-NTA chromatography under denaturing conditions and refolded (Figure 2A). The refolded GBNV NSm, devoid of membrane was used for analysis of its interaction with liposomes. The membrane floatation assay and analytical ultracentrifugation confirmed that refolded NSm can associate with membranes (Figure 2B and C).

The Far UV CD spectrum of the refolded NSm (Figure 3A) was different from the spectrum of the protein purified by sucrose density gradient (Figure 1C). Further, addition of liposomes (Figure 3A) or SDS (Figure 3C) at submicellar concentration resulted in increase in ellipticity at 220 and 208 nm as observed earlier for TMV MP [3] and P7 of Carmovirus [8]. The intrinsic fluorescence spectrum of refolded NSm showed emission maximum at 341 nm (Figure 3B) and addition of liposomes resulted in quenching of fluorescence suggesting that membrane association could alter the orientation of aromatic residues.

*In planta* expressed NSm could not be solubilised by addition of triton X100, 4 M urea or Na_2_CO_3_ suggesting that NSm is either an integral membrane protein or it forms large aggregates. In the absence of transmembrane domain, it is possible that NSm forms large aggregates via interaction through the coiled coil domain *In* *planta*. These aggregates are not solubilised by triton X100 or 4 M urea (Figure 6).

Surprisingly the deletion of C-terminal coiled coil domain resulted in the loss of membrane association by NSm (Figure 5D). It was shown earlier that the VAP (Virion Associated Protein) of CaMV interacts with movement protein via coiled coil region which is indispensable for the viral infection [54]. However, the coiled coil domains are not present in the MPs of all the plant viruses. The presence of a conserved coiled coil domain in the NSm protein might be important for Tospoviruses, the movement and encapsidation of which are different as compared to positive single stranded RNA plant viruses. Membrane floatation assay of C-terminal deletion mutant confirms its role in membrane association (Figure 5C). It was reported earlier [28], by alanine scanning mutation in TSWV NSm that mutations at A269–274 led to the loss of tubule formation and cell to cell movement. But the exact mechanism of loss of cell to cell movement could not be elucidated. It is possible that loss of coiled coil domain in this region might be responsible for loss of movement function.

Yet another protein, the REMORIN1.3 protein of *Solanum tuberosum,* a plasmodesmata residing protein, was shown to anchor itself to membrane via its C-terminal coiled coil domain [55]. Additionally there are several reports on the movement proteins hijacking endocytic pathways for their localization to plasmodesmata. Therefore, it appears that NSm might use the C-terminal coiled coil domain for vesicle formation which is targeted to plasmodesmata. Further experiments are required to prove this hypothesis. This would be of physiological relevance as the protein might increase the size exclusion limit of plasmodesmata by flattening the membranes and there by facilitate cell to cell movement of the virus.
